# Temporal sequence learning in winner-take-all networks of spiking neurons demonstrated in a brain-based device

**DOI:** 10.3389/fnbot.2013.00010

**Published:** 2013-06-06

**Authors:** Jeffrey L. McKinstry, Gerald M. Edelman

**Affiliations:** The Neurosciences InstituteSan Diego, CA, USA

**Keywords:** neurorobotics, sequence learning, spiking network, winner-take-all, motor control and learning/plasticity, spike-timing dependent plasticity, sensorimotor control, large-scale spiking neural networks

## Abstract

Animal behavior often involves a temporally ordered sequence of actions learned from experience. Here we describe simulations of interconnected networks of spiking neurons that learn to generate patterns of activity in correct temporal order. The simulation consists of large-scale networks of thousands of excitatory and inhibitory neurons that exhibit short-term synaptic plasticity and spike-timing dependent synaptic plasticity. The neural architecture within each area is arranged to evoke winner-take-all (WTA) patterns of neural activity that persist for tens of milliseconds. In order to generate and switch between consecutive firing patterns in correct temporal order, a reentrant exchange of signals between these areas was necessary. To demonstrate the capacity of this arrangement, we used the simulation to train a brain-based device responding to visual input by autonomously generating temporal sequences of motor actions.

## Introduction

A growing body of neurophysiological evidence suggests that patterns of activity in vertebrate brains observed during movement are commonly composed of temporal sequences of periods with steady-state firing rates lasting several hundred milliseconds separated by sharp transitions (Tanji, 2001; Averbeck et al., 2002; Nakajima et al., 2009). This pattern of activity is also observed during sensory perception in gustatory cortex (Jones et al., 2007), and the operation of working memory (Seidemann et al., 1996). Although network models composed of mean-firing-rate neurons have been used to model sequential neural activity (Rhodes et al., 2004; Salinas, 2009; Verduzco-Flores et al., 2012), biological networks are composed of spiking neurons. Therefore, understanding spiking networks with this capability requires further exploration (Liu and Buonomano, 2009; Chersi et al., 2011). Given open questions regarding the stability and robustness of networks which learn to generate sequences (Verduzco-Flores et al., 2012), testing such networks in Brain-Based-Devices (BBD) is warranted (Edelman, 2007; McKinstry et al., 2008).

In this paper we describe how our previous models of Winner-Take-All (WTA) spiking networks (Chen et al., 2013) can be coupled together and trained to generate segmented and sequential neural activity (see Rutishauser and Douglas, 2009 for a mean-firing rate WTA network that generates sequences). The neural system is composed of thousands of simulated biologically realistic excitatory and inhibitory spiking neurons. The single compartment neurons modeled in these simulations display voltage dynamics similar to those seen in cortical neurons. Activity of the simulated neurons reflects the conductance of ion channels in the model including: AMPA, NMDA, GABAa, and GABAb (Izhikevich and Edelman, 2008). Model synapses were subject to short-term synaptic plasticity (Zucker, 1989). Spike-timing dependent plasticity (STDP) modeled long-term synaptic changes that allowed the system to learn temporal sequences.

We found that networks composed of spiking neurons of this sort, when trained to respond to repeated sequences of sensory cues, generate temporally ordered patterns of neuronal activity consisting of brief steady states separated by sharp transitions that resemble those observed in functioning brains. We found that the present model could be used to control specific motor sequences in a brain-based device. The population activity pattern in this modeled neuronal system has similarities to those observed in primate pre-frontal cortex during multi-segmented limb movements (Averbeck et al., 2002).

## Materials and methods

### Spiking neuronal networks

**Figure 1:**
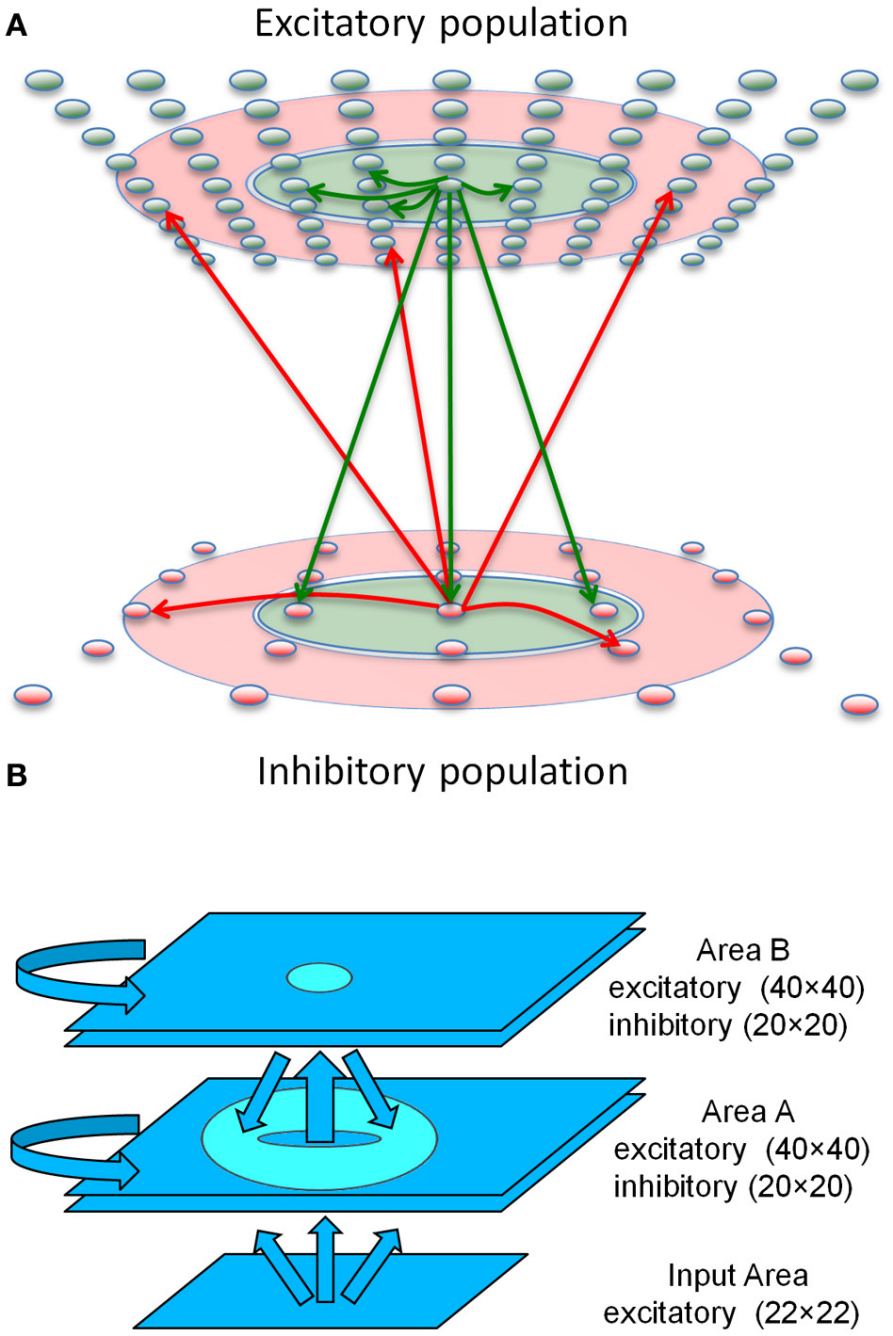
**Sequence generation network architecture. (A)** Center-Annular-Surround (CAS) network architecture that produces WTA dynamics. The CAS network architecture consists of interconnected spiking neurons, excitatory (green ovals) and inhibitory (red ovals). Each population is arranged in a two-dimensional grid. Connections from representative cells are illustrated. Axons from excitatory neurons (green arrows) project to neurons within green areas. Axons from inhibitory neurons (red arrows) project to neurons in the transparent red annular areas. The CAS connectivity leads to WTA dynamics, in which small regions of high activity are surrounded by large regions with little activity. **(B)** The sequence generation network is comprised of two reentrantly interconnected Center-Annular-Surround (CAS) spiking networks, Areas A and B. Arrows indicate directions of neural connectivity, while the circle and the donut shape indicate the inter-network connectivity (projection field) from single points in the projecting network. The input area projects non-topographically to Area A. Area A projects topographically to Area B, as indicated by the small oval in Area B. In turn, Area B projects topographically and widely back to area A, but not to the same spot from which it received input, as indicated by the donut-shaped ring in Area A. Avoiding projections to the corresponding spot helped prevent the network from locking into a single activity pattern due to self-amplification. Rather it allowed the network to switch smoothly between patterns in a sequence.

Each modeled network (Figure 1A) is comprised of up to three interconnected populations of spiking neuronal units (Izhikevich, 2010) distributed over two-dimensional square grids. Each population is composed of units simulating one of three functional classes of spiking neurons: input, excitatory, and inhibitory. The parameters of simulated neurons in each class are tuned so that the voltage waveform mimics its biological counterpart. The synapses display STDP and short-term plasticity dynamics as previously described in detail (Izhikevich and Edelman, 2008). The neuron model equations, short-term synaptic plasticity equations, and STDP equations are presented after a description of the network connectivity.

### Neuronal network architecture

Each of the three major structural and functional components of the modeled nervous system (Figure 1B) consisted of a network of spiking neuronal units (Izhikevich) distributed over a two-dimensional (2 mm by 2 mm) grid. The networks function as analogs of a thalamic nucleus (Input area), and two interconnected cortical areas (Area A and Area B).

The Input network contained 484 simulated neurons providing topographic excitatory input to Area A. Current levels to cells of the Input area were adjusted by trial and error to assure that the network responded to abstract patterns or video camera input by generating distinct response patterns of neuronal activity with a maximum firing rate of ~100 Hz. The Area A and Area B networks were each made up of 1600 excitatory cells as well as 400 inhibitory cells having fast-spiking behavior.

Areas A and B had similar connectivity. Each was composed of a Center-Annular-Surround (CAS) network, a variant of center-surround networks, that we have found (Chen et al., 2013) to effectively generate WTA dynamics (Dayan and Abbott, 2001) in large-scale networks of spiking neurons. Any distinctive pattern of neural activity in the input area evoked enhanced neural activity within a few localized patches in both areas A and B. This CAS network architecture is illustrated in Figure 1A. Connectivity between the model neurons fell into two classes: either local-type or surround-type. Local-type connections are between nearby neighbors, whereas surround-type connections come from neighbors in a surrounding annular region. Excitatory cells receive both local-type projections from excitatory cells and surround-type inhibitory projections (Figure 1A). Inhibitory cells also received local-type projections from the excitatory cells and surround-type input from other inhibitory cells. The CAS connectivity confers WTA properties to both areas A and B. A complete description of all connectivity parameters is provided in the Supplementary Material.

To create a network capable of storing and generating sequences of neural activity, we added reentrant connections between Areas A and B in the following way. In Area A (Figure 1B), both excitatory and inhibitory cells also receive simulated feed-forward input that was approximately all-to-all. Area B neurons, on the other hand, do not receive connections from the input. Instead, they receive non-plastic, local-type input that is topographic from Area A. Area B excitatory neurons project back to Area A with plastic and widespread surround-type connectivity. Synaptic changes resulting from STDP at these connections form a link between temporally adjacent patterns of neural activity within the sequence. These excitatory reentrant connections from Area B to Area A are widespread and cover most of the region since each activity pattern in Area B has two bumps, similar to the activity pattern shown in Figure S5. This widespread connectivity enables the network to learn to associate arbitrary temporally adjacent patterns. This was useful for the BBD experiment, since the patterns that emerged within Area A during the initial training phase were not under experimenter control.

### Neuronal dynamics

Spiking dynamics of each neuron were simulated using the phenomenological model proposed by Izhikevich (2003). The model has only 2 equations and 4 dimensionless parameters that could be explicitly found from neuronal resting potential, input resistance, rheobase current, and other measurable characteristics. We present the model in a dimensional form so that the membrane potential is in millivolts, the current is in picoamperes and the time is in milliseconds:

(1) $$C\dot{v}=k(v-v_{r})(v-v_{t})-u-I_{\text{syn}}$$

(2) $$\dot{u}=a\left\{b(v-v_{r})-u\right\}$$

where *C* is the membrane capacitance [in picofarads (pF)], *v* is the membrane potential (in mV), *v*_*r*_ is the resting potential (in mV), *v*_*t*_ is the instantaneous threshold potential (in mV), *u* is the recovery variable (the difference of all inward and outward voltage-gated currents in pA), *I*_syn_ is the synaptic current (in pA) defined below, *a* and *b* are unitless parameters. When the membrane potential reaches the peak of the spike, i.e., *v* > *v*_peak_, the model is said to fire a spike, and all variables are reset according to *v* ← *c* and *u* ← *u* + *d*, where *c* (mV) and *d* (pA) are parameters. Table SI lists each of the neuron model parameters used in all experiments. At the start of all simulations, *v* was set to –60 for all neurons, whereas *u* was set to a different random value for each neuron, drawn uniformly from the range 0–100.

### Short-term synaptic plasticity

The strength of synapses varied as a function of the pre-synaptic neuron's recent firing history independent of long-term synaptic changes as found in biological synapses (Zucker, 1989). We assume that the synaptic conductance (strength) of each synapse can be scaled down (depression) or up (facilitation) on a short time scale (hundreds of milliseconds) by a scalar factor *x*. This scalar factor, different for each pre-synaptic cell, is modeled by the following one-dimensional equation

(3) $$\dot{x}=\left(1-x\right)/\tau_{x},\;x\leftarrow px\text{ when pre-synaptic neuron fires.}$$

*x* tends to recover to the equilibrium value *x* = 1 with the time constant τ_*x*_ (in ms), and it is reset by each spike of the pre-synaptic cell to the new value *px*. Any value *p* < 1 decreases *x* and results in short-term synaptic depression, whereas *p* > 1 results in short-term synaptic facilitation. The parameters, τ_*x*_, in ms, and scale factor *p*, for each combination of pre-synaptic and post-synaptic neuron type were as follows: exc.→ exc.: 150, 0.8; exc. → inh.: 150, 0.8; inh. → exc.: 150, 0.8; inh. → inh.: 150, 0.8; thalamic → exc.: 150, 0.7; thalamic → inh.: 200, 0.5.

### Synaptic kinetics

The total synaptic current to each neuron is simulated as,

(4) $$\begin{array}{l} I_{syn}=g_{AMPA}(v-0)+g_{NMDA}\frac{{\left[(v+80)/60\right]}^{2}}{1+{\left[(v+80)/60\right]}^{2}}(v-0)\\ \;+g_{NMDA_{VI}}\frac{{\left[(v+100)/60\right]}^{2}}{1+{\left[(v+100)/60\right]}^{2}}(v-0)\\ \;+g_{GABA_{A}}(v+70)+g_{GABA_{B}}(v+90) \end{array}$$

where *v* is the post-synaptic membrane potential, and the subscript indicates the receptor type. Each millisecond, each synaptic conductance is updated according to Equation 5.

(5) $$g_{r}(t)=\{\begin{array}{l} g_{r}(t-1)-g_{r}(t-1)/\tau_{r}+{\text{gain}}_{r}\;xs(t-1)\text{ when the}\\ \text{ presynaptic neuron fires}\\ g_{r}(t-1)-g_{r}(t-1)/\tau_{r}\text{ otherwise} \end{array}$$

where subscript *r* indicates the receptor type, τ_*r*_ = 5, 150, 6, and 150 ms for the simulated AMPA, NMDA, GABA_A_, and GABA_B_ receptors, respectively. The voltage-independent NMDA channel (NMDA_VI_) is based loosely on the type of channel found between excitatory cells in layer 4 of visual cortex (Binshtok et al., 2006); we used τ_*r*_ = 150 ms for this simulated receptor as well. *s*(*t*) is the synaptic weight at time *t*. *x* is the short-term depression/potentiation scaling factor as above; gain_NMDA_ is the ratio of NMDA to AMPA conductance and is found experimentally to be less than one (Myme et al., 2003). Similarly, gain_GABAB_ is the ratio of GABAB to GABAA receptors. The values of gain_AMPA_ and gain_GABAA_ were always one. The values of gain_NMDA_ and gain_GABAB_ used in the simulations are shown in Tables SII, SIII.

### STDP

The long-term change in conductance (weight) of each synapse in the model is simulated according to STDP: the synapse is potentiated or depressed depending on the order of firing of the pre- and post-synaptic neurons (Bi and Poo, 1998). We use the following equations to update the state of each plastic synapse, *s*(*t*), in the network every millisecond:

(6) $$y(t)=y(t-1)-y(t-1)/\tau_{c}+\alpha \text{STDP}(t)\delta (t-t_{\text{pre/post}})$$

(7) $$s(t)=\{\begin{array}{l} s(t-1)+y(t)\text{ if mod}(t,50)=0\\ s(t-1)\text{ otherwise} \end{array}$$

where δ(*t*) is the Dirac delta function that step-increases the variable *y*. Firings of pre- and post-synaptic neurons, occurring at times *t*_pre_, *t*_post_, respectively, change *y* by the amount αSTDP(*t*) where α is the learning rate for the synapse, *t* = *t*_post_ − *t*_pre_ is the interspike interval, and

(8) $$\text{STDP}(t)=\left\{\begin{array}{l} A^{+}\text{exp}{\left(-1/\tau^{+}\right)}^{t},t>0\\ A^{-}\text{exp}{\left(-1/\tau^{-}\right)}^{\left|t\right|},t\leq 0 \end{array}\right\}.$$

where *A*^+^ = 0.005, *A*^−^ = 0.001, τ^+^ = τ^−^ = 20 ms. The variable *c* decays to 0 exponentially with the time constant τ_*c*_ = 1000 ms. Each synapse is updated only once every 50 ms for computational efficiency. Note that for simplicity, each synapse was modeled with a single weight, *s*; therefore the STDP rule changed both AMPA and NMDA components of the synapse proportionally. In addition, each synapse was prevented from exceeding *s*_max_ or going below 0, regardless of learning rules and normalization (see synaptic scaling). Values of *s*_max_ for each connection pathway are provided in Tables SII, SIII.

### Synaptic scaling

Synaptic strengths at time *t*, *s*^*j*^_*i*_ (*t*), were scaled for each synapse *i*, in order to maintain the total of all synaptic strengths on a given connection pathway to neuron *j*, *s*_total_, at a constant value:

$$s_{i}^{j}(t)=s_{i}^{j}(t-1)\left(\frac{s_{\text{total}}}{\sum_{k\;=\;1}^{n_{j}}s_{k}^{j}(t-1)}\right)$$

where *n*_*j*_ is the number of synapses on the connection pathway to neuron *j*. This scaling was performed for every neuron each time the synapses were updated with Equation 7. Values of *s*_total_ for each connection pathway are provided in Tables SII, SIII.

### Data analysis

To evaluate how accurately the network regenerated individual activity patterns within a sequence, we calculated the similarity between the network response to each individual segment (pattern), and the population response during sequence training and recall. To measure similarity between two neural activity patterns in a given population at two different times, t1 and t2, the following steps were performed. The mean firing rate of each neuron in the population at time t1 was calculated within some small window, yielding a number for each neuron; this list of numbers formed a vector, **f1**. The same was done at time t2, yielding vector **f2**. A match score was computed between the two population vectors by taking the normalized dot-product as follows:

$$\text{match}=\frac{\sum_{i\;=\;1}^{n}f1_{i}\cdot f2_{i}}{\left\|\bar{f1}\right\|\left\|\bar{f2}\right\|}$$

where *n* is the number of neurons in the population, and $\left\|\bar{x}\right\|$ computes the length of the vector $\bar{x}$. This match score provides a measure of similarity where one is a perfect match, and 0 is a complete mismatch.

The mean firing rate of each Area A excitatory neuron in response to each input stimulus in the sequence was recorded during the first epoch of sequence training during which there was no overlap in the input patterns presented or in the corresponding network responses to those patterns. Subsequently, during sequence training and free recall phases, these templates were used to quantify how closely an observed pattern of neural activity resembled each individual segment of a sequence. To do this, the mean firing rate vector of Area A neurons was computed every 50 ms of sequential behavior. A match score was then calculated between each of the sub-pattern templates and the template of each 50-ms population firing rate segment. This method can detect whether ongoing spiking activity reflects multiple sub-patterns of a sequence at the same time. It makes no assumptions about the time-course of sequence generation.

### Brain-based device

**Figure 2:**
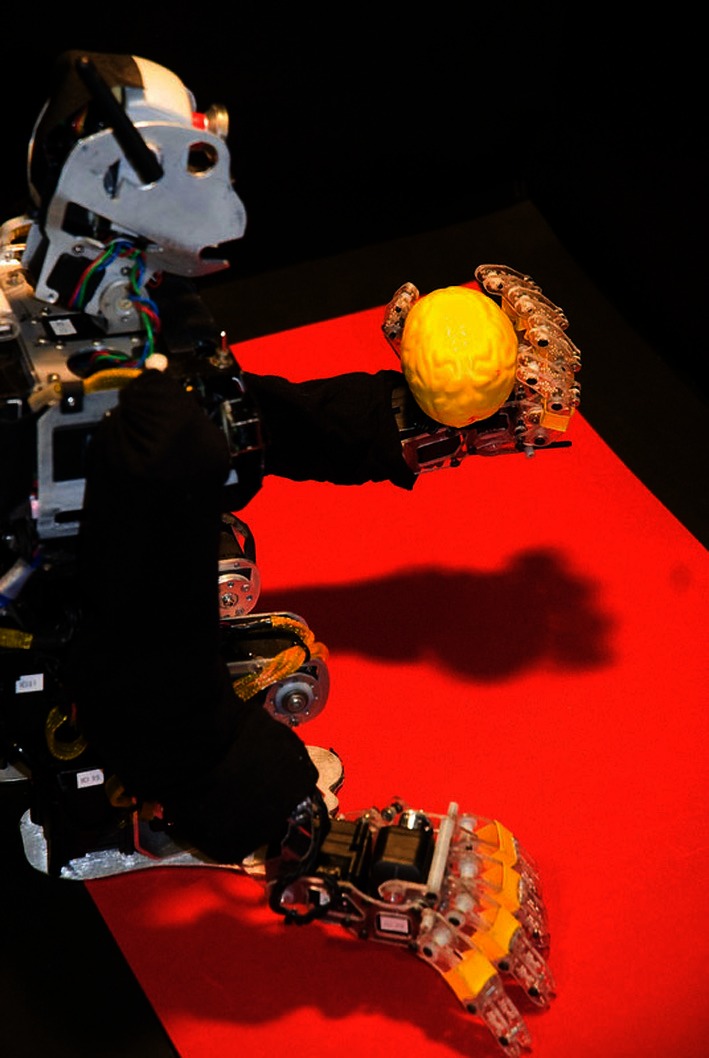
**Custom humanoid robot, or brain-based device, used for behavioral tests of the sequence generation network**. The BBD has a grayscale camera which monitors the location of the bright object in its hand in order to learn “hand-eye” coordination of its left hand. During experiments the left arm was moved repeatedly in a sequence of four different postures. See Materials and Methods for a detailed description of the device.

To investigate a simulated nervous system in a real-world device, we designed and constructed a humanoid BBD (Figure 2). This device is ~20 inches high and uses a black and white wireless webcam for vision. Each arm contains eight Dynamixel motors (Robotis, Irvine, CA, USA). In the experiments described here only the two shoulder motors function; all other joints remain stationary with the arm extended. Shoulder joint angles provided by the motors determine the posture of the arms. A miniature PC (VIA Technologies, Fremont, CA, USA) mounted on the back of the BBD-maintained wireless communication between the device and the spiking neuronal networks simulated on a Mac-Pro (Apple, Inc. Cupertino, CA, USA). The robot operated ~3 times slower than real-time during experiments.

To test the sequence generation network in the BBD, a motor area in addition to area A and B was added to enable the system to generate motor sequences (see Figure S1). This network was the same size as the excitatory-inhibitory networks in Areas A and B, with 1600 excitatory, and 400 inhibitory spiking neurons, and had similar parameters as well. Different patterns of spiking of motor area neurons specified distinct equilibrium postures of the left arm using population vector coding as described in the Supplementary Material. Since the video camera was aimed at the robotic hand and remained fixed during the experiments, each of these postures, in turn, evoked a distinct pattern of visual input to the system. The motor region received non-topographic connections from the output of Area B in the sequence generation network. These connections were also subject to STDP and homeostatic plasticity, which allowed arbitrary sensorimotor transformations to develop during training. A more detailed description of the network along with the parameter settings used in the experiments can be found in the Supplementary Material.

## Results

### Simulated neural activity during temporally segmented behavior

**Figure 3:**
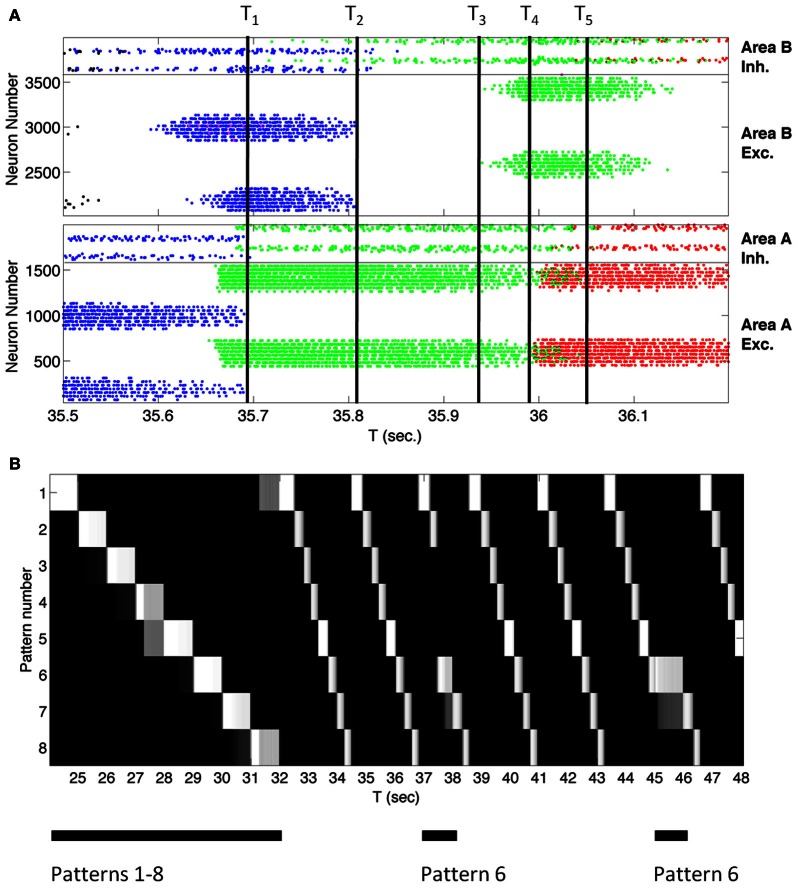
**A large-scale network of ~4000 spiking neurons autonomously transitions between states reflecting a learned sequence. (A)** Spike rastergram of all neurons in Networks A and B showing the population activity recorded over 700 ms. as the network spontaneously generated the learned sequence. Each spike is shown as a colored dot and each neuron is assigned a color to indicate the pattern to which it responds maximally. Networks A and B transitioned spontaneously between stable-states corresponding to three learned input patterns in the sequence as indicated by the three colors blue, green, and red associated with patterns four, five, and six, respectively. [The few magenta dots are associated with neurons responding best to another pattern, but which are also activated by pattern four (blue)]. The four neural populations in the network are labeled on the right of the diagram. Inh., Inhibitory; Exc., Excitatory. The labels T_1_ through T_5_ mark transition times referred to in the text. **(B)** After training, the coupled networks spontaneously generate a sequence of eight patterns in the correct order, and that sequence can be interrupted or shifted by presenting an external stimulus. Each row of the figure indicates, by brightness, the match score over time for one of the eight patterns that make up the sequence. The match score for pattern number X for example indicates how closely the neural population activity pattern in the Area A excitatory neurons matched the activity in the same population recorded when pattern X was presented to the network for the first time during training. White is a perfect match, while black indicates a complete mismatch. The network was trained from *t* = 0 to 32 s by stimulating the eight patterns in order. The times of stimulus presentation are indicated by black bars under the figure. The internally generated sequence is interruptible. When presented with one pattern in the sequence, pattern 6, for 1 s at *t* = 37 s and *t* = 45 s, the network activity immediately reflected the stimulus, and when the stimulus was removed the network generated the sequence from pattern 6 onward.

Before describing the BBD experiment, we illustrate the capability of the sequence network to learn to generate sequences of simulated responses to sensory inputs. The system of reentrantly coupled CAS networks can learn to reproduce a temporal sequence of eight consecutive input patterns. These individual patterns were simulated by means of current injections into Area A excitatory cells (see Figure S2 for resulting network activity patterns). Multiple presentations of a given temporal sequence to the network constituted a training regimen. A given sequence consisted of an ordered series of eight distinct, randomly generated input patterns. Each pattern was presented for 1 s. After 32 s of training, the input was discontinued. At this point, the network continued to regenerate the eight patterns in correct order. Figure 3A shows a raster plot of the spiking of all excitatory and inhibitory neurons in the simulation as the system autonomously cycled through the trained sequence for 700 ms. The pattern of activity of the neuronal population consisted of a series of stable microstates—periods in which each neuron fires at a steady rate—each lasting ~100 ms, flanked by briefer, more complex transition states.

A brief account of the mechanisms by which the network develops sequence generation ability will aide in understanding what follows. One way to form a network that recognizes and generates temporal sequences of input patterns is to establish serial connections between distinct neuronal groups. If each neuronal group responds to a different input pattern—due to WTA dynamics—and a sequence of unique patterns is presented, then the neuronal groups will be activated successively. Given sufficient temporal overlap between the activity in successively responding neuronal groups, Hebbian mechanisms will act to strengthen the connections between them. These connections favor activation of the next neuronal group in the sequence in the absence of the external input, allowing for internal pattern generation of an arbitrary temporal sequence learned through experience. Separating the network into two populations, Area A reflecting the current pattern, and Area B reflecting the prior pattern, allows simultaneous activity in temporally adjacent neuronal groups, one in each WTA area, facilitating synaptic change via Hebbian learning.

Figure 3A illustrates the mechanisms underlying the microstate transitions between neuronal group activations within a temporal sequence after training. At the time labeled T_1_ in Figure 3A, the activity in area A that reflected pattern 4 (blue dots) ceased. Active neurons in area B no longer received input and ceased to fire at time T_2_ when voltage-*independent* NMDA currents, which characterize this network, decayed. Due to the loss of lateral inhibition, neurons in Area B giving rise to pattern 5 (green) began to fire at time T_3_ in response to input from area A. Once these Area B cells for pattern 5 were activated, they triggered the firing of cells in Area A that correspond to pattern 6 (red) at time T_4_. At time T_5_, cells in Area A corresponding to pattern 5 no longer received input from Area B and ceased to fire. The network continued to advance through a series of microstates in this fashion until all patterns were generated.

Figure 3B reflects an analysis of spiking data from this simulated network, acquired over a longer period, 24 s. Each row in the figure plots the match score (in 50 ms time bins) to one of the eight training patterns. White is a perfect match, while black indicates a complete mismatch. The last training repeat is from *t* = 24 to *t* = 32. Subsequently, external stimulation was removed and STDP was discontinued in order to test whether training was successful. Nevertheless network activity continued autonomously. After presenting any one pattern in the sequence, the network repeatedly cycled through the patterns until another input stimulus was presented. Because the network had been presented with repeated transitions from pattern 8 to pattern 1 during training, the network cycled through all eight patterns repeatedly until it was interrupted. In order to test that these results were reproducible, the simulation was performed five times in total using different pseudorandom number seeds from the standard C library (Kernighan and Ritchie, 1988) to distribute the initial synaptic connectivities and strengths in the networks; in every case the system recalled eight patterns in the correct order.

**Figure 4:**
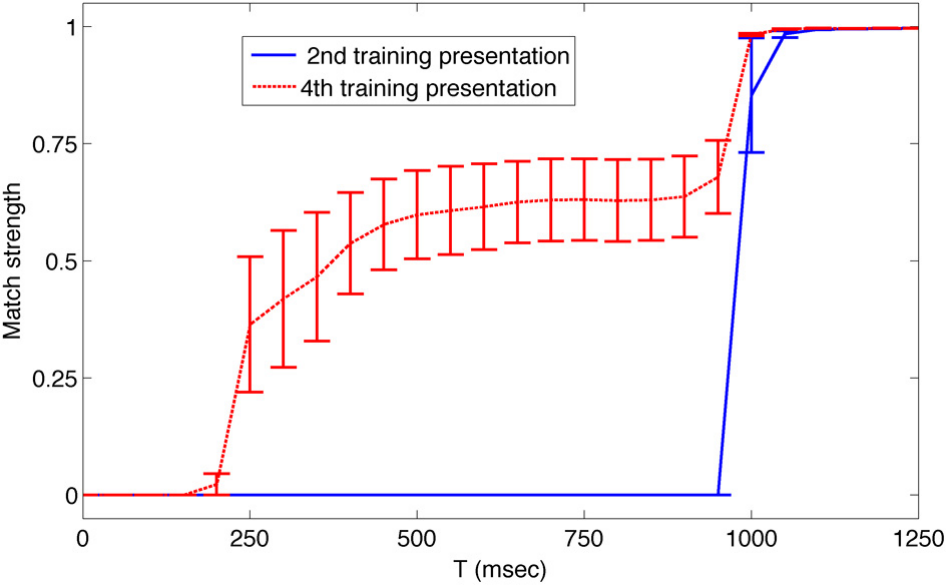
**After training, the simulated neural system anticipates upcoming patterns in the sequence**. The average match strength of individual patterns in the sequence is plotted over time during the presentation of the *prior* pattern. Data was obtained during the second presentation of the sequence from *t* = 9 to *t* = 16 s (blue solid line), and during the fourth presentation of the sequence from *t* = 25 to *t* = 32 s (red dashed line). During the fourth presentation, 250 ms after each new pattern was presented, the activity of neurons in Area A began to match that of the next pattern in the sequence. Error bars show the standard error of the mean match response.

Although the system of networks repeatedly regenerated the learned sequence autonomously, it nonetheless remained responsive to novel external input. To demonstrate this, we interrupted the autonomous activity every 8 s by presenting the input corresponding to a different member of the set of learned patterns. For example, as shown in Figure 3B at *t* = 37 s, pattern 6 was presented out of order for 1 s to reset network activity. Subsequently the sequence continued in the trained order. Thus, after being presented with a repeated series of input patterns, this system of networks correctly anticipated the next pattern in a temporal sequence. Figure 4 shows plots of the average match score of each pattern in a sequence during the 1 s presentation of the previous pattern. During the second presentation of the sequence from *t* = 9 to *t* = 16, the match score is 0 (blue solid line), but during the fourth training trial from *t* = 25 to *t* = 32 the match score to the anticipated pattern increases after 250 ms (red dashed line), indicating that the system has formed an association between temporally adjacent patterns in the sequence. Similar results were obtained in all five simulations with different initial conditions.

### Motor control of a BBD

**Figure 5:**
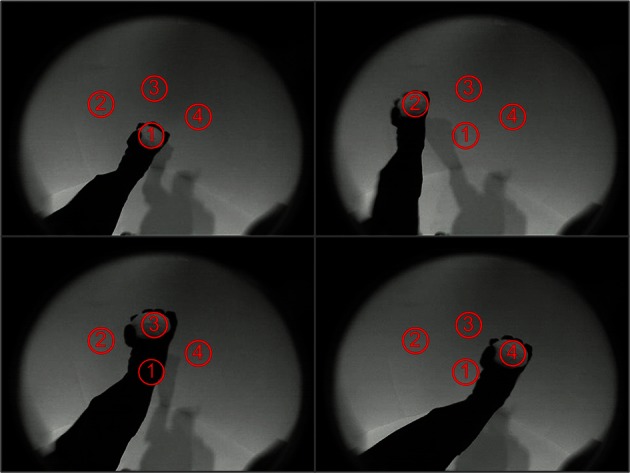
**The four arm postures of the BBD as viewed through its video camera**. The BBD was trained to move its hand consecutively in numerical order to the 4 spots outlined in red. The images show all four arm postures of the BBD. During training, a bright object placed in the hand made the hand positions salient against the dark background (The lighting was increased when these images were taken to provide sufficient contrast to see the arm). During a test for spontaneous recall of the learned sequence, the bright object was removed to eliminate visual input.

To demonstrate the use of this system of simulated neuronal networks to regulate real-world behavior in real-time, the spiking output of the sequence generation network was used to control three-dimensional movements of a BBD. The task for the device was to learn to move its hand autonomously in a pre-specified order through four different locations in its visual field. Visual input from the BBD's camera was used to drive a retina model that projected topographically to the Input area of the sequence generation network. This allowed the BBD to learn a sequence of visual stimuli (see Supplementary Material for details of the retina model). The BBD was placed in a seated position with its camera looking toward its left side. A bright object was placed in its hand to provide salient stimulation at the location of the BBD's left hand in the visual field. Figure 5 shows examples of the raw video input from the camera with the arm in each of the four postures used in the experiment. To generate the desired sequence of segmented arm movements, the pattern of spiking excitatory cells of Area B was used as input to the simulated motor network. To establish the hand-eye coordination that this task requires, two angles of the right shoulder of the robot were successively manipulated to position its hand for 1 s in each of the four locations in the visual field. This was accomplished by injecting appropriate current into groups of neurons within the simulated motor area. This was repeated a total of 5 times. During this first training stage from *t* = 1 to *t* = 20 s, this system learned to discriminate between the four different spatial visual patterns. STDP was activated on the pathway from the Input area to Area A. The CAS network operating in Area A developed sparse activity patterns discriminating these four Input area patterns (Chen et al., 2013) (see Figures S3–S5 for example of activity patterns from one simulution). Non-plastic topographic connections from Area A to Area B essentially create a copy of Area A's pattern of activity in Area B.

A second training stage was used to allow the system to learn hand-eye coordination. The stimulation patterns from stage one were repeated from *t* = 21 to *t* = 40 s while STDP was activated on the pathway from Area B to the motor area. During this stage, this system came to associate the visual responses in Area B evoked by different postures with the pattern of motoric output that generated and maintained these postures (Figure 5). After this training stage, hand-eye coordination was established, but sequence learning had not yet been achieved.

**Figure 6:**
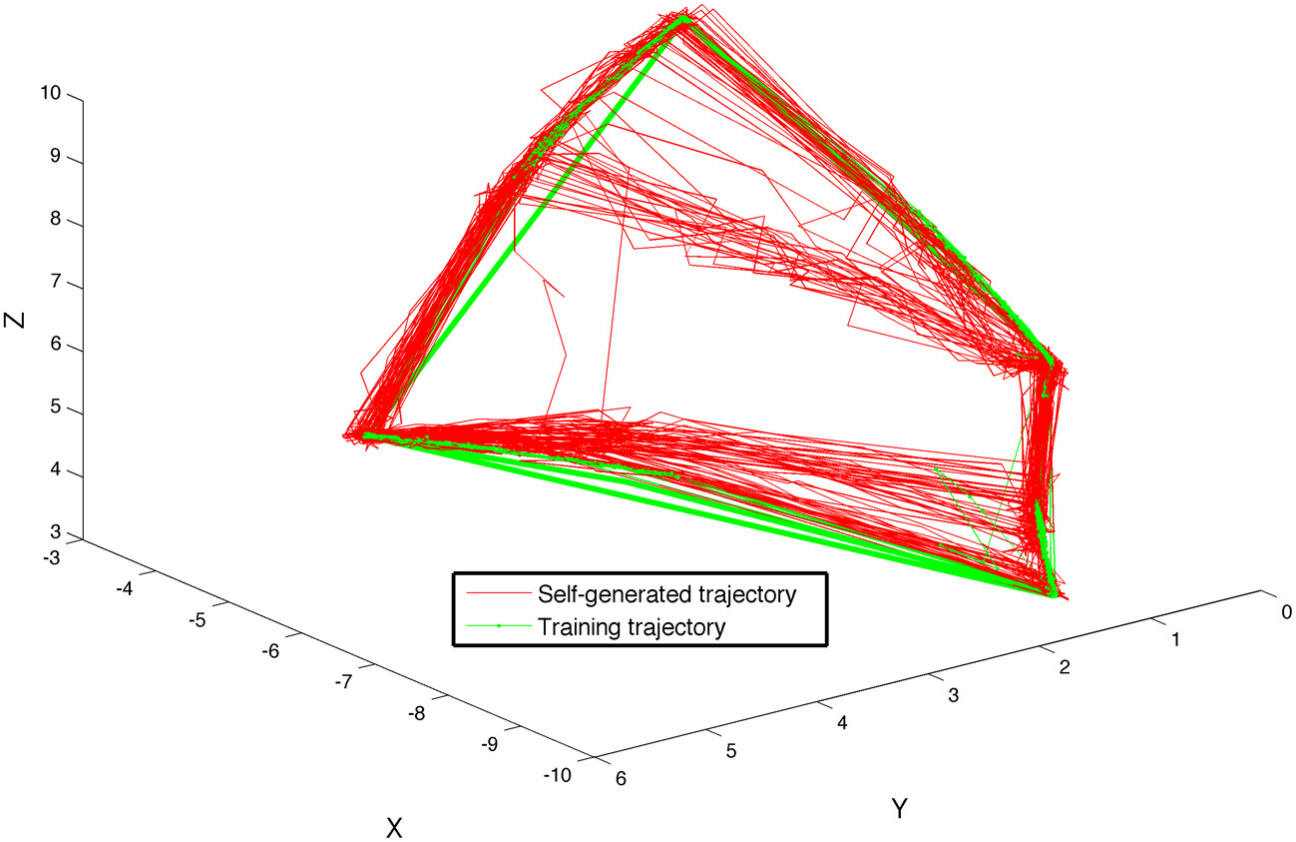
**The BBD self-generates a learned sequence of arm motions using the sequence generation network model**. The BBD was trained to move its arm through four different postures such that its hand traced out a quadrilateral in space. The figure shows the superposition of hand positions recorded during 20 s of training (green line) and during 20 s of self-generated movements after training (red line) for all 5 subjects. Lines are drawn between temporally adjacent data points recorded at 50 ms intervals. One of the five subjects showed some error and consistently “cut” the upper corner, generating a different shape than the other four subjects. The self-generated arm trajectories approximate the training trajectories.

A final training stage was used to train the visual sequence network to generate the sequence of visual patterns corresponding to a sequence of arm movements. During this stage, from *t* = 41 to *t* = 60 s, STDP was activated on the pathway from Area B to area A for the first time. The system was trained by moving the arm of the device once again five times through the sequence of four postures, pausing 1 s at each posture. Subsequently, after the camera input to the system was discontinued, the BBD continued to autonomously generate motor commands that evoked movements similar to those used during the training phase. Each segment of the autonomous gestures lasted ~400 ms, the experiment was performed five times incorporating different initial network parameters. Each time it reproduced the correct continuous sequence of movements. Figure 6 shows a trace of the movements made by the hand of the BBD during the five experiments, plotted in Cartesian coordinates both during the last training stage (green), and for 20 s after training during autonomous motor sequence generation (red). Positions were calculated from joint angles recorded every 200 ms during the simulation. One of the five subjects showed some error and consistently “cut” the upper corner, generating a different shape than the other four subjects. The self-generated arm trajectories approximate the training trajectories.

We verified that the system remained responsive to external visual stimulation while it continued to generate the trained sequence autonomously. For each of the five subjects, visual stimulation was resumed at *t* = 100 s. While the BBD continued to cycle through the trained sequence, the bright object used for training was moved sequentially by the experimenter to each of the locations in space it occupied during training and was held in place from 3 to 10 s. In 19 of 20 trials (five subjects tested at four locations) the BBD moved its hand to the location of the object and held it there until the experimenter removed the object from the visual field, at which point the BBD resumed the learned sequence from its present location. In one trial, the BBD moved its hand to the object, but then resumed the sequence prior to stimulus removal.

## Discussion

The robust recognition and regeneration of motor sequences known to occur in animals is accomplished by networks of spiking neurons. Here we show that this basic capability can be simulated using large-scale networks of spiking neurons. The computational model employed here can be further elaborated to explore sequence recognition and generation in networks consisting of groups of reentrantly connected neurons. The simulations demonstrate how networks composed of thousands of densely interconnected spiking neurons can respond adaptively to patterned sensory input by generating autonomous, temporally-ordered sequences of neural activity. We found that the operation of STDP to shape the distribution of synaptic strengths within and among WTA networks can give rise to network responses able to control complex behavior in a robotic device.

The system described here builds upon our previous work with large-scale spiking networks (Chen et al., 2013). The prior work explored the use of WTA networks for visual pattern categorization and feed-forward mappings between a sensory and motor map. The prior system was not capable of learning sequences. The present work demonstrates that coupling two WTA spiking networks together with specific reentrant connections leads to the ability to regenerate sequences after experience. The prior system was entirely sensory driven, while the present work allows internally generated network activity in the absence of sensory input (Figure 3), yet remains responsive to external input. Further, the rapid microstate transitions observed in this network are consistent with cortical microstate transitions.

Several theoretical models of behavioral sequence generation have been reported in the literature. Rhodes et al. (2004) proposed a mean-firing-rate model, N-STREAMS, which reproduces the physiological results of Averbeck et al. (2002). Salinas studied a mean-firing-rate simulation that incorporates rank-order-selective (ROS) neurons into a network and showed that the model could learn sequential motor actions given such neural responses. The activity of the ROS neuronal units was built-into the model, and did not emerge through learning. Verduzco-Flores et al. (2012) created a small mean-firing-rate network with 200 neurons that could learn multiple sequences with shared subsequences. Their model required temporally adjacent input patterns to partially overlap in time. Finally, Chersi et al. (2011) investigated a spiking network model that generates chains of temporal sequences of neural activity similar to those in our model and comparable to neurophysiological responses found in the intra-parietal lobe in primates. They used four separate pools of 500 neurons each to represent one of four different actions. Sparse connections between the 4 pools were subject to STDP. They showed that repeated activation of the neurons in the 4 pools in a given temporal order via simulated current injection eventually lead to correct recall of the remaining sequence after injecting only the first pattern. Our model does not require the use of discrete pools of neurons; rather such pools emerge automatically within each network through a WTA competition in the CAS architecture.

It is interesting to consider wither there is a benefit to using spiking neurons instead of rate-based neurons in the brain-based device. The sequence generation network may have worked just as well with a model incorporating mean-firing rate neurons. Nevertheless it is important to demonstrate that spiking networks can generate such behavior, because animal nervous systems incorporate spiking neurons. This work demonstrates that spiking networks incorporating STDP can be reliably trained to generate sequences in the real-world.

By using simulated neuronal networks to control the behavior of a BBD we found that a real world device can be trained to generate autonomous, multi-segmented behavior. After training the system by presenting the target pattern of video input in 1-s time steps, the BBD regenerates this sequential input pattern, but at a faster rate. The BBD was able to recreate movements composed of four consecutive steps in the correct order. Although the device can remember and reproduce multiple sequences of behavior, each posture within any sequence must be unique. Otherwise the subsequent posture would be ambiguous. Learning more complex behaviors will require incorporation of longer temporal contexts than those provided by the immediately preceding pattern.

The spiking activity corresponding to consecutive equilibrium postures in the behavioral sequence overlap in time, similar to activity reported in primates (Averbeck et al., 2002). This can be seen in Figure 3B from second 24 to 32. For example, shortly after the network responds with a high match score to input pattern 3 at *t* = 26, network activity begins also to match pattern 4. The match scores (shades of gray) to each subsequent pattern begin to increase well before the pattern is presented to the network. In primates, this overlap in neural responses reflects current and future gestures made by the animal as it draws shapes “in the air.” Averbeck et al. (2002) also reported that the neural activity pattern corresponding to the current gesture was more strongly represented than the activity pattern reflecting the upcoming movement. This behavior is seen in our network. The match score for the current pattern, pattern 3, in Figure 3 at *t* = 26 is higher than the match score for the upcoming pattern 4.

Over time, spiking activity in the model network transits through a series of microstates, each characterized by a stable unique pattern of steady-state firing rates (Figure 3A). Similar behavior has been observed in mammalian cortex. For example, neurons in the gustatory cortex in rodents (Jones et al., 2007), and in the pre-frontal cortex of primates (Seidemann et al., 1996) progress through sequences of states, identifiable in examinations of simultaneously recorded neuronal ensembles. As in the simulation reported here, these states lasted for hundreds of milliseconds, with rapid transitions on the order of 50 ms. Our model suggests that such microstate transitions may be explained as reentrant interactions (Edelman, 1978) between multiple WTA networks.

### Conflict of interest statement

The authors declare that the research was conducted in the absence of any commercial or financial relationships that could be construed as a potential conflict of interest.
