# Supplementary material for: Temporal sequence learning in winner-take-all networks of spiking neurons demonstrated in a brain-based device
Source: Front Neurorobot. 2013 Jun 6;7:10. doi: 10.3389/fnbot.2013.00010 (PMC3674315; doi:10.3389/fnbot.2013.00010)
Supplement: Supplementary file 1 [file DataSheet1.PDF]

Supplementary Material.

Neuron parameters

Table I shows each of the neuron model parameters used in all experiments.

Table I. Neuronal parameters. a.u.: arbitrary units.

Neuron type	Area	C (pF)	k (a.u.)	v_r (mV)	v_t (mV)	v_{peak} (mV)	a (a.u.)	b (a.u.)	c (mV)	d (pA)
Exc.	A	80	3	-60	-50	50	0.01	5	-60	10
Inh.	A	20	1	-55	-40	25	0.15	8	-55	200
Exc.	Motor	100	0.7	-60	-50	0	0.03	-2	-60	100
Inh.	Motor	20	1	-55	-40	25	0.15	8	-55	200
Exc.	B	80	3	-60	-50	50	0.01	5	-60	10
Inh.	B	20	1	-55	-40	25	0.15	8	-55	200
Thalamic	Input	200	1.6	-60	-50	40	0.01	15	-60	10

Anatomy.

The connectivity between model neurons fell into two classes: either local-type or surround-type. For local-type connectivity, a two-dimensional Gaussian probability distribution, centered on each post-synaptic cell, determined the probability of forming a synapse between each potential pre-synaptic neuron within a specified maximum distance, r_{max}

$$f(d) = ae^{-\frac{(d-\mu)^2}{2\sigma^2}} \qquad (8)$$

where *a* is a scale factor set to generate, on average, a target number of synapses on each post-synaptic cell, *d* is the distance between the pre-synaptic neuron and the post-synaptic neuron, μ is 0, and σ is the standard deviation. In a similar manner, a two-dimensional Gaussian function was also used to specify the synaptic strength between connected neurons as a function of the distance between them in the network. The total of all synaptic efficacies was scaled to sum to a constant parameter with units in nanoSiemens (nS). Both connection probability and strength were maximal between nearest neighbors, and fell off as a function of distance, controlled by the same parameter, the standard deviation of a Gaussian.

For surround-type connectivity, a post-synaptic neuron receives synaptic connections from neurons located in a surrounding annular region specified by a minimum (r_{min}) and maximum (r_{max}) radial distance from the post-synaptic cell. (This is equivalent to saying that each pre-synaptic neuron sends projections to post-synaptic neurons in an annular region). The probability of forming a connection with a neuron in the annulus is

determined as a function of distance from the post-synaptic cell. The function used is a Gaussian with standard deviation σ, centered at $\mu=(r_{min}+r_{max})/2$. Thus a post-synaptic neuron does not connect with a neuron within the central ring, has minimal connection probability at the inner ring of the annulus, maximal probability half-way between the inner and outer ring of the annulus, and no connections beyond the outer ring. This probability density function is scaled to create a prespecified number of synapses for each post-synaptic neuron. The synaptic strengths for the surround-type connection are also initialized using the same function, with the same parameters. However, the sum of all synaptic strengths of this type was scaled to make the total equal to a constant value under experimenter control.

In order to avoid boundary conditions in the network, the network was treated as a torus. Thus connections from neurons that would go outside of the network instead "wrap around" to connect with neurons on the opposite edge.

Table II shows the parameters defining the anatomy and synaptic parameters of the sequence generation network for the network with simulated sequence inputs. The table defines two types of information for every neural area: the neuron composition, and the synaptic connectivity for each neuron type. The first four columns of the table list, for each separate neural population in the simulation, the type of neuron, the area in which they are located, the number of neurons in the population, and the total number of synapses per neuron.

The remaining columns define the connectivity for each type of neuron in the area. Multiple rows are necessary to define the connectivity for each post-synaptic type; one row is needed for each presynaptic neuron type forming synapses on the post-synaptic neurons. Pre-area and pre-type specify the presynaptic area and type of a neuronal group projecting to the post-synaptic group. The next column specifies the percentage of the post-synaptic cell's synapses allocated to this pathway. The remaining columns provide all of the parameters used to specify details of the synaptic pathways as described in the paragraphs above.

Table III show the parameters defining the anatomy and synaptic parameters of the motor sequence network used to control the arm of the BBD; the format is the same as Table II.

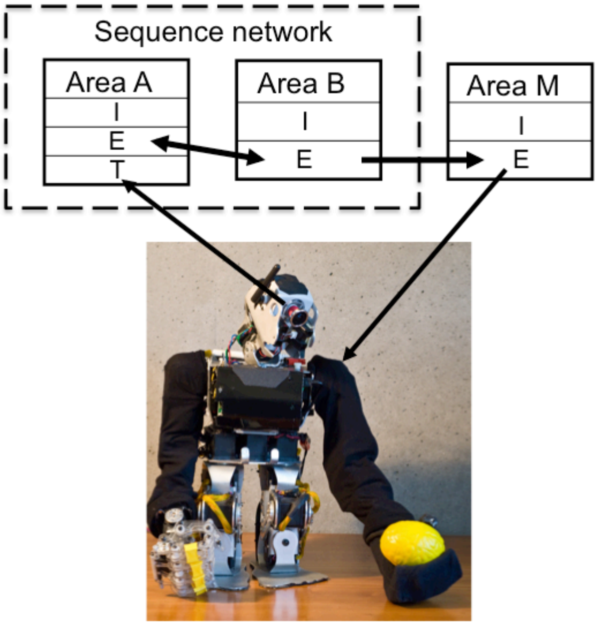

Supplementary Figure 1. The neural architecture for the BBD experiments.

Neural architecture for the BBD experiment.

Supplementary Figure 1 illustrates the neural architecture used for the BBD experiments. The sequence network consists of two CAS networks, Area A and B, each consisting of populations of excitatory (E) and inhibitory (I) neurons synaptically coupled as described in the paper. Visual input from the video camera provided patterned input to "thalamic" (T) neurons of Area A, while the output of excitatory neurons in the motor area (M) were used to control the two shoulder joints of the left arm. After repeatedly stimulating the motor area in one of four different locations in the same order, and thus moving the arm to one of four different postures, a mapping formed from the visual area responses to the location of the hand to the motor area output that drove the hand to those locations. The sequence of four visual patterns was learned by the sequence network, Areas A and B. As this network recalled the visual sequence after training, associated area M activity caused the arm to move to the posture associated with the next visual pattern in the sequence, as shown in Figure 6.

Mapping motor network activity to arm postures.

In order to translate from neuronal firings to joint angle in the left shoulder of the BBD, the output of the motor network cells was pooled using a form of population vector averaging described next.

Each excitatory neuron of the motor network, Area M, was assigned a preferred set of angles for each of the two shoulder joints. Nearby neurons in this predetermined map evoked similar joint angles, but different patterns of activity among these cells could evoke all possible positions of the left arm. Neuron *i* at grid position *u,v* in the network prefers shoulder angles $(w_i^1, w_i^2) = (u/40, v/40)$.

For each joint, *j*, the preferred joint angles of all cells, weighted by the corresponding firing rate, were added together to determine an equilibrium posture as follows:

$$angle_j = \begin{cases} \dfrac{\sum_{i=1}^{n} w_i^j r_i}{\sum_{i=1}^{n} r_i}, when \geq 10\ neurons\ have\ r_i > 15\ hz \\ \alpha_j, otherwise \end{cases},$$

where w_i^j is the angle for joint *j* preferred by cell *i*, and r_i is the estimated firing rate of the cell estimated with a time constant of 100 ms. Joint angles were recalculated in this manner, and shoulder joints were adjusted every 250 milliseconds.

Visual input.

Video was recorded with an Axis 207MW wifi camera. Grayscale images with a resolution of 320x240 were transmitted at 30fps. The pixels from the central portion of the video frames were used to mimic retinal photoreceptors. These pixels provided input to a two-dimensional grid of on-center Retinal Ganglion Cells (RGC). The grid size was 21x21 neurons with a center area receptive field size of 3x3 and the surround area of 6x6 pixels. Each RGC receives a current that is computed following the algorithm of Wohrer and Kornprobst (2009). These currents were constantly injected at each integration step until the next video frame was received. RGCs were modeled with the Izhikevich model (Izhikevich and Edelman, 2008) with the following parameters: C=100, Vr=-70mV, Vt=-50mV, k=1, a=0.005, b=0, c= -75mV, d=250, and Vpeak=10mV.

Training stimulation patterns used in the initial sequence experiment.

The first experiment uses eight artificial stimulation patterns to train the sequence network. We assume that each population pattern corresponds to a unique network response to an external sensory input. Supplementary Figure 2 plots the 8 individual population firing rate patterns resulting from current stimulation into a subset of the Area A excitatory neurons. Within each diagram, the color of each pixel indicates the firing rate of one neuron in the population, as indicated by the color scale to the right of each diagram. Each simulated neuron with a non-zero firing rate was stimulated with a current injection of 1,000 pA for one second. These eight patterns were present one at a time and repeated in the network in the sequence shown in the figure from right to left, top to bottom.

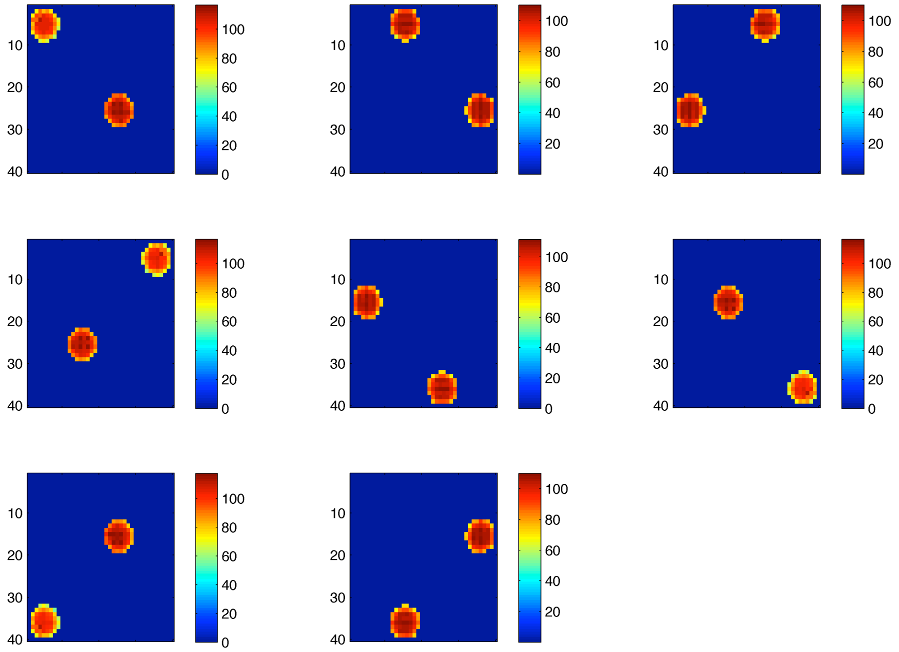

Supplementary Figure 2. The simulated population activity patterns used to train the sequence network. See supplementary text for details.

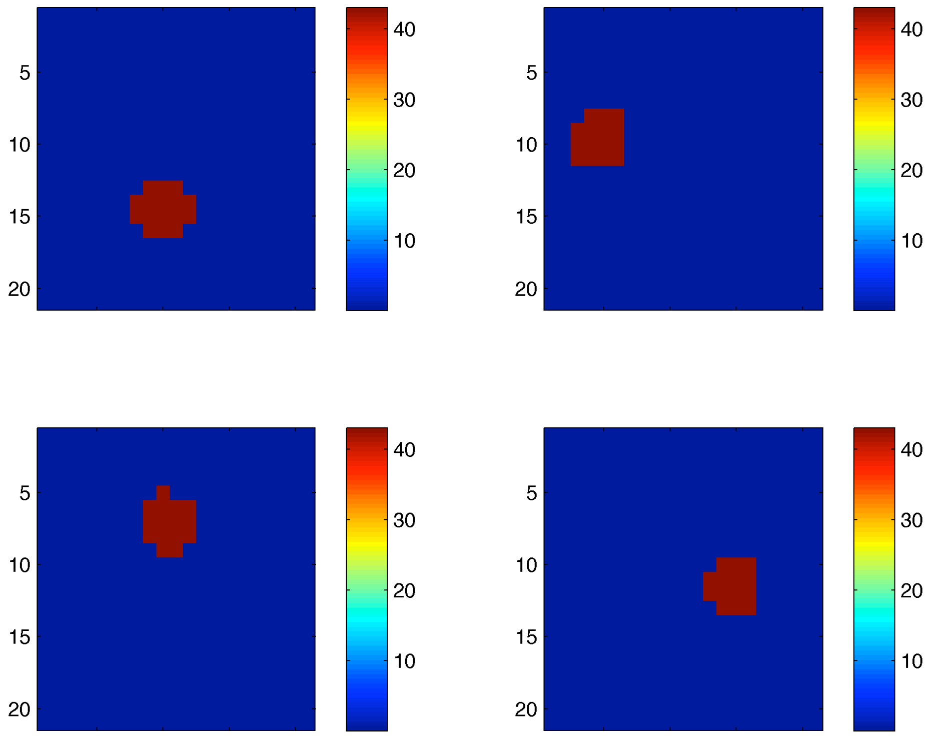

Supplementary Figure 3. The four population activity patterns in the Input area used in the first training stage in the BBD experiment from *t*=1 to *t*=20 simulation seconds (the network runs slower than real-time, and we measure time in network simulation cycles with a time step of 1ms). Plots are similar to supplementary figure 2. These four firing rate patterns reflect the visual patterns from the grayscale camera sensing the hand of APE-X in four different locations during 1 experiment. The color scale to the right of the four map indicates the mean firing rate associated with each color.

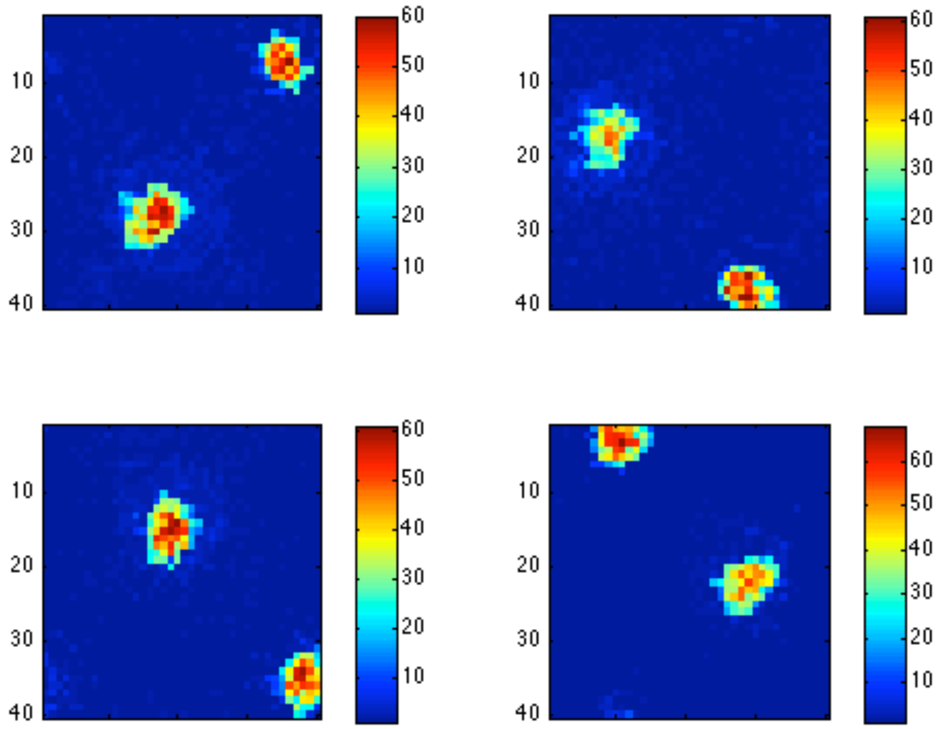

Supplementary Figure 4. The four population activity patterns in area A in response to the four stimulus patterns shown in Supplementary Figure 3 the first time they are presented, from t=1 to t=4s for one BBD subject. Color scale to the right indicates the mean firing rate associated with each color.

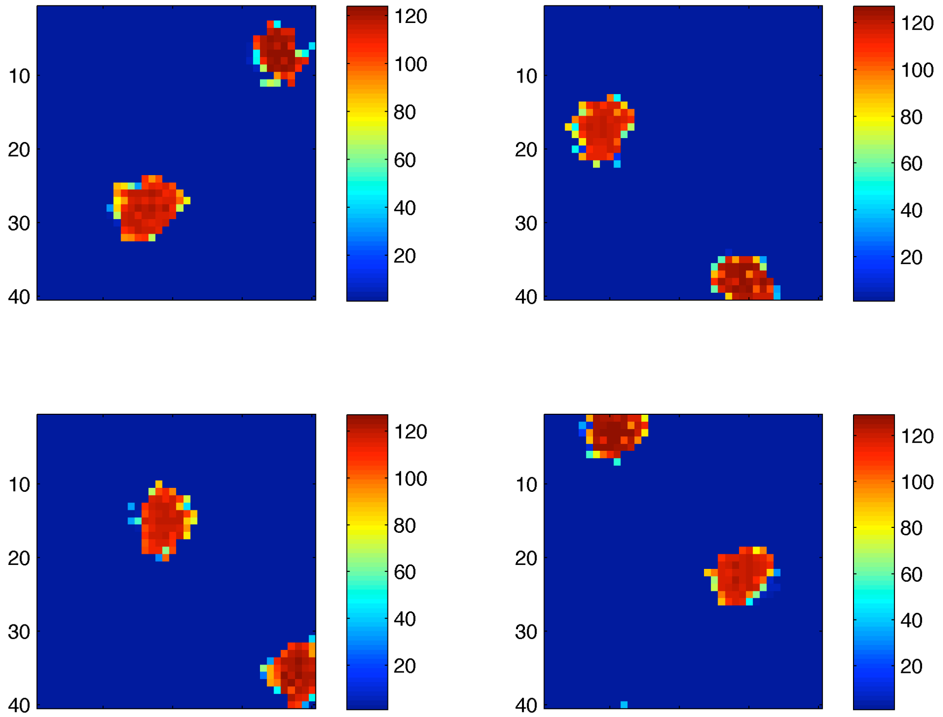

Supplementary Figure 5. Similar to Supplementary Figure 4 but showing population responses during the fifth presentation of each pattern, from t=16 to t=19s. The increased firing rates are due to STDP acting on connections from the Input area to excitatory cells in Area A, and local connections between excitatory cells within Area A.

References

Izhikevich EM and Edelman GM (2008). Large-scale model of mammalian thalamocortical systems. Proc Natl Acad Sci U S A. 105(9):3593-3598.

Wohrer A, Kornprobst P (2009). Virtual Retina: a biological retina model and simulator, with contrast gain control. Journal of Computational Neuroscience 26(2), 219–249.

Table II. Anatomical and synaptic parameters for the sequence generation network used in experiments with simulated input.

Post-synaptic neuron type	Post-synaptic area	Number of neurons	Average synapses per neuron	Pre-synaptic area	Pre-synaptic neuron type	Percentage of total synapses	r_{min} (mm)	r_{max} (mm)	σ (mm)	ε	s_{total} (nS)	s_{max} (nS)	Initial α	Final α	Learning start time (ms)	Learning end time (ms)	$gain_{NMDA}$	$gain_{NMDAVI}$	$gain_{GABAB}$
Exc.	A	1600	3080	A	Exc.	14	-	0.1	0.05	0	22	10	0	0	0	0	0.5	0	0.1
				A	Inh.	29	0.1	1	0.8	0	1200	20	0	0	0	0	0.5	0	0.1
				B	Exc.	29	0.36	1	0.3	0	60	30	0.9	0.9	0	32000	0	0.5	0.1
				Input	Thalamic	29	-	1.44	2.5	0	200	50	0.9	0.9	0	32000	0.5	0	0.1
Exc.	B	1600	2200	B	Exc.	40	-	0.15	0.07	0	30	5	0.9	0.9	0	32000	0.5	0	0.1
				B	Inh.	40	0.15	1	0.3333	0	900	10	0	0	0	0	0.5	0	0.1
				A	Exc.	20	-	0.15	0.07	0	22	5	0	0	0	0	0.5	0	0.1
Inh.	A	400	2000	A	Exc.	20	-	0.33	0.16	0	25	5	0	0	0	0	0.5	0	0.1
				A	Inh.	40	0.15	1	0.3333	0	180	3	0	0	0	0	0.5	0	0.1
				Input	Thalamic	40	-	1.44	10	0	15	10	0	0	0	0	0.5	0	0.1
Inh.	B	400	2000	B	Exc.	20	-	0.33	0.16	0	15	2	0	0	0	0	0.5	0	0.1
				B	Inh.	40	0.15	1	0.3333	0	180	3	0	0	0	0	0.5	0	0.1
				A	Exc.	40	-	0.15	100	0	15	50	0	0	0	0	0.5	0	0.1
Thalamic	Input	484	0	-	-	0	-	0	0	0	0	0	0	0	0	0	0.5	0	0.1

Table III. Anatomical and synaptic parameters for the sequence generation network used in experiments with the BBD.

Post-synaptic neuron type	Post-synaptic area	Number of neurons	Average synapses per neuron	Pre-synaptic area	Pre-synaptic neuron type	Percentage of total synapses	r_{min} (mm)	r_{max} (mm)	σ (mm)	ε	s_{total} (nS)	s_{max} (nS)	Initial α	Final α	Learning start time (ms)	Learning end time (ms)	$gain_{NMDA}$	$gain_{NMDAVI}$	$gain_{GABAB}$
Exc.	A	1600	3080	A	Exc.	14	-	0.1	0.05	0	22	10	0	0	0	0	0.5	0	0.1
				A	Inh.	29	0.1	1	0.8	0	1200	20	0	0	0	0	0.5	0	0.1
				B	Exc.	29	0.36	1	0.3	0	60	30	0.9	0.9	40000	60000	0	0.5	0.1
				Input	Thalamic	29	-	1.44	2.5	0	200	50	0.9	0.9	0	20000	0.5	0	0.1
Exc.	B	1600	2200	B	Exc.	40	-	0.15	0.07	0	30	5	0.9	0.9	0	20000	0.5	0	0.1
				B	Inh.	40	0.15	1	0.3333	0	900	10	0	0	0	0	0.5	0	0.1
				A	Exc.	20	-	0.15	0.07	0	22	5	0	0	0	0	0.5	0	0.1
Inh.	A	400	2000	A	Exc.	20	-	0.33	0.16	0	25	5	0	0	0	0	0.5	0	0.1
				A	Inh.	40	0.15	1	0.3333	0	180	3	0	0	0	0	0.5	0	0.1
				Input	Thalamic	40	-	1.44	10	0	15	10	0	0	0	0	0.5	0	0.1
Inh.	B	400	2000	B	Exc.	20	-	0.33	0.16	0	15	2	0	0	0	0	0.5	0	0.1
				B	Inh.	40	0.15	1	0.3333	0	180	3	0	0	0	0	0.5	0	0.1
				A	Exc.	40	-	0.15	100	0	15	50	0	0	0	0	0.5	0	0.1
Thalamic	Input	484	0	-	-	0	-	0	0	0	0	0	0	0	0	0	0.5	0	0.1
Exc.	Motor	1600	1800	A	Exc.	33	-	0.33	0.16	0	5	5	0.9	0.1	20000	40000	0.5	0	0.1
				A	Inh.	45	0.3333	1.444	10	0	1200	100	0	0	0	0	0.5	0	0.1
				B	Exc.	22	-	1.414	4.5	0.5	20	5	0.9	0.1	20000	40000	0.5	0	0.1
Inh.	Motor	400	1000	A	Exc.	60	-	0.33	0.16	0	10	2	0	0	0	0	0.5	0	0.1
				A	Inh.	40	0.3333	1.444	10	0	800	20	0	0	0	0	0.5	0	0.1
